# Practical Anatomy: A New Arrangement of the London Dissector, with Numerous Modifications and Additions, Containing a Concise Description of the Muscles, Bloodvessels, Nerves, Viscera and Ligaments of the Human Body, as They Appear on Dissection

**Published:** 1856-11

**Authors:** 


For sale by PHINNEY & Co.

---

ART. V.—*Practical Anatomy:* a new Arrangement of the London Dissector, with numerous modifications and additions, containing a concise description of the muscles, bloodvessels, nerves, viscera and ligaments of the human body, as they appear on dissection. With illustrations. By D. HAYES AGNEW, M. D., Lecturer on Anatomy and Surgeon to the Philadelphia Hospital. Philadelphia: J. B. LIPPINCOTT & Co. 1856.

This little volume has considerable merit as a dissector. We are not familiar with it, and cannot speak authoritatively of its merits, compared with Wilson's or the Dublin Dissector.

For sale by PHINNEY & Co.

---

*Mrs. Partington on Baths.*—"This is a great discovery, to be sure," said Mrs. Partington, with animation, "when people that have experienced salvation through calumny and all sorts of pisenous grediencies, can have it soaked out of 'em." We asked what she meant, and looked at her as she sat in meditation on the little low chair in the corner, revolving the idea, which pressed upon her brain, like a weight of steam, two hundred and fifty pounds to the square inch. "Why," said she, smiling like the moon with reflection, "there is a contrivance for soaking a man who has taken calumny and minerals all his lifetime, till his joints are as stiff as wooden legs in the last war, and when he comes out of the bath and wipes himself with a hacmetic towel, he has n't a single mineral in him—he is a perfect vegetable, as limber as an eel!" What a gratified look it was she gave, as an imaginary procession of cripples, the victims of calomel, passed before her mind's eye, like the spirits of Kossuth's countrymen, as she thought of their leaping all cured from the bath!

